# The Medial Sural Artery Perforator Free Flap: A Novel, yet Challenging and Versatile Flap for Head and Neck Reconstruction

**DOI:** 10.7759/cureus.32572

**Published:** 2022-12-15

**Authors:** Aseem Mishra, Abhishek Das, Gautam Prakash, Monika Gupta, Nachammai Nagrajan

**Affiliations:** 1 Head and Neck Surgery, Mahamana Pandit Madan Mohan Malaviya Cancer Centre, Varanasi, IND; 2 Plastic Surgery, Institute of Medical Sciences, Banaras Hindu University, Varanasi, IND; 3 Prosthodontics, Institute of Medical Sciences, Banaras Hindu University, Varanasi, IND

**Keywords:** oral cavity soft-tissue reconstruction, head and neck reconstruction, microsurgery free flaps, medial sural artery perforator flap, free flap transfer

## Abstract

Background

In head and neck reconstruction, especially after cancer ablation, choosing the best flap is critical. Due to its thin, lengthy, and malleable vascular pedicle as well as low donor site morbidity, the medial sural artery perforator (MSAP) free flap is gaining popularity among reconstructive surgeons, particularly in oral soft-tissue reconstructions. The goal of this study was to record the clinical use of an MSAP flap in the repair of post-oncologic lesions in the head and neck region.

Methodology

Patients with oral cancer who came to our center after ablative surgery on the buccal mucosa, tongue, floor of the mouth, and hard palate were repaired utilizing the MSAP flap. Preoperatively, the perforators were discovered using an 8 MHz portable doppler and a computed tomography angiogram. Without the use of a tourniquet, the flaps were delineated and harvested. In all of our patients, a single venous anastomosis was sufficient.

Results

The MSAP flap was used to positively rebuild 14 cancer patients, with the flap design based on the amount of the resection site or defect. The buccal mucosa (n = 7) and tongue (n = 6) were the most common subsites of the ablative defect. The average flap size was 12 × 6 cm, with a thickness of 5-7 mm. In eight cases, the donor site defect was mostly closed, with the remaining patients receiving split-thickness grafts for subsequent healing. In 12 cases, the best cosmetic and functional results were obtained. One patient developed a postoperative wound infection, and the flap could not be saved. Another patient developed a neck hematoma which had to be debrided on the second postoperative day, with good results. In primary closure cases, patients were mobilized with full weight-bearing on the first postoperative day.

Conclusions

MSAP flap is an alternative to radial forearm free flap and anterolateral thigh flap in obese patients with medium-sized oral abnormalities. This flap stands out as an outstanding option for head and neck soft-tissue reconstruction due to its unique mix of flap thinness, greater skin region, and superior donor site cosmesis. Despite its importance, just a few case studies and reports have been published. A multicenter trial with a high sample size would demonstrate the use of this flap and its chimeric designs.

## Introduction

One of the most common reasons for head and neck reconstruction is post-oncological resection. Extensive analysis for facial reconstruction should be made [1]. Complex flaws and a limited local donor option due to the aggressive removal of the tumor are two factors that influence the reconstruction of a head and neck cancer patient. Microsurgical free flap reconstruction is the most common method for restoring function in the head and neck, and perforator flaps are gaining popularity due to their versatility.

Perforator-based fasciocutaneous flaps, which are sustained by perforating vessels, capture the same skin area as musculocutaneous flaps. They are gaining in popularity and far outnumber other options. It leaves the muscle reasonably intact, allowing the function to be preserved while reducing donor-site morbidity.

The medial sural artery perforator (MSAP) flap, first described by Cavadas et al. in 2001 [2], has some unique characteristics that make it a better choice in certain situations. It combines the pliability of radial forearm free flaps with the low donor-site morbidity of anterolateral thigh flaps. It has a potentially lengthy vascular pedicle, and the flap is quite small even in obese patients, making it a viable alternative to the similarly thin radial forearm flap. This could be due to the steep learning curve and higher complication rates. Despite the concerns raised by this flap’s persistent nature, a recent meta-analysis identified the MSAP tissue transfer as an excellent candidate for head and neck reconstructions [3-5].

The goal of this article is to share our expertise and the approach we employed to harvest MSAP flaps, as well as the intraoperative findings and patient follow-up with functional outcomes. We addressed the advantages and disadvantages of MSAP flaps as our preferred alternative for head and neck reconstruction in cancer patients with few issues encountered during the recovery period.

## Materials and methods

From December 2019 to February 2022, our center conducted a retrospective analysis of 14 patients who underwent resection of head and neck tumors and underwent MSAP flap reconstructions after a joint clinic decision. This study was performed as a retrospective case series, and, therefore, approval from the regional ethical committee was not necessary. All patients provided written and informed consent prior to surgery. The study’s goal was to share our experience with the flap in soft-tissue intraoral reconstructions. The demographics and tumor factors were studied. Details such as flap size, resection extent, and flap features, such as the number of perforators detected, location, and pedicle length, were recorded throughout the procedure. The overall duration of the operation was also recorded. Resections were performed by a single surgeon with adequate margins (>1 cm) grossly. In case of doubt, the frozen section was used to confirm microscopic spread beyond margins. Reconstruction of the defect was performed by a single surgeon to reduce inter-operator variability. Closure of the donor site (either primary or skin grafts) was documented. Any flap failures or long-term complications such as hypertrophic scarring, itching, pigmentation, or functional impairment were recorded. Small-to-medium-sized soft-tissue abnormalities, particularly in obese people, were included in the study. Free MSAP flap transfer was not recommended for patients with major lower leg damage, peripheral vascular disease, or diabetes.

Surgical anatomy

The medial sural artery and vein arise at the level of the knee joint and are branches of the corresponding popliteal arteries. Intramuscular branching patterns are diverse, making intramuscular dissection challenging. These veins travel a few centimeters before entering the hilum at the undersurface of the medial gastrocnemius muscle. The major trunk of the vascular pedicle runs parallel to the fibers of the gastrocnemius muscle. Although the exact number of useable musculocutaneous perforators in the medial gastrocnemius muscle varies widely, every individual in this zone has at least one large perforator [4-6].

Preoperative evaluation

In a standing position, the patient’s lower limbs were assessed. By pressing the skin together to assess for primary closure of the donor site, the pliability of the tissues was determined. For preoperative examination, a handheld Doppler and computed tomography (CT) angiogram were used. Two lines were drawn from the midpoint of the popliteal crease to the tendoachillies and a third line from the midpoint of the popliteal crease to the medial maleolus.

Harvesting procedure

**Figure 1:**
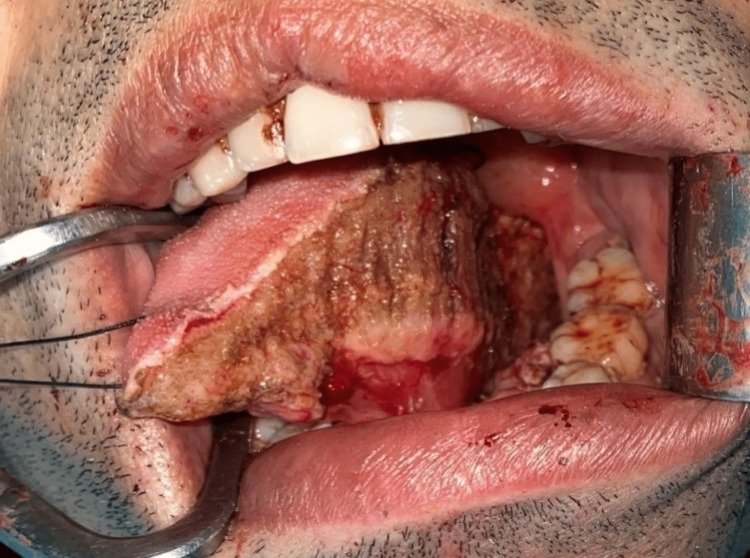
Intraoperative defect of the tongue: hemiglossectomy + floor of mouth resection.

**Figure 2:**
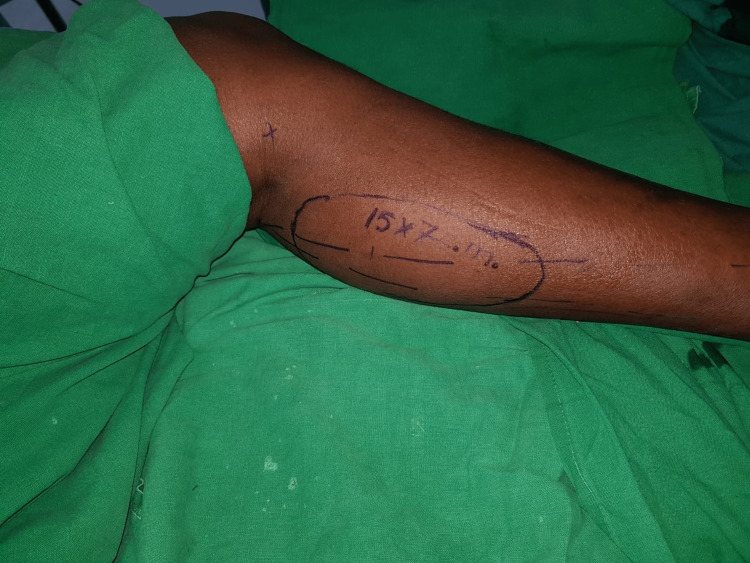
Anatomical landmarks of the medial sural artery perforator flap.

**Figure 3:**
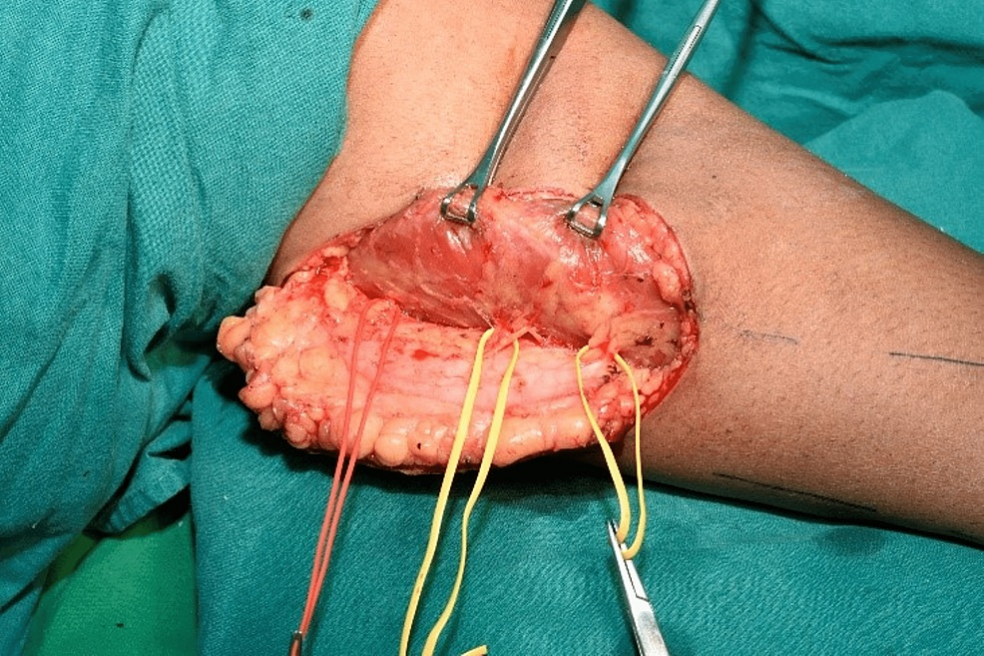
Intraoperative view of the harvested medial sural artery perforator flap.

**Figure 4:**
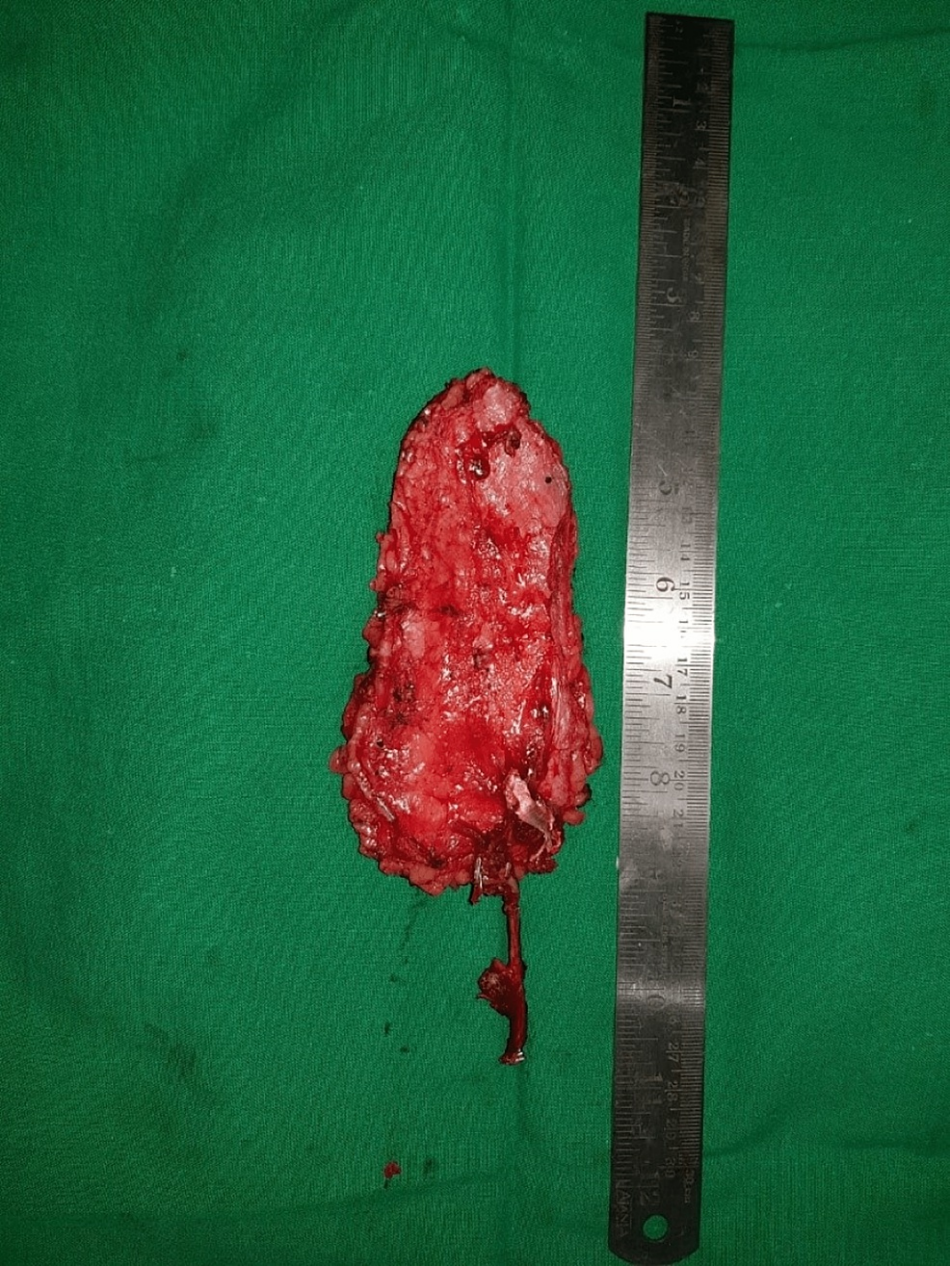
Intraoperative view of the flap before inserting.

**Figure 5:**
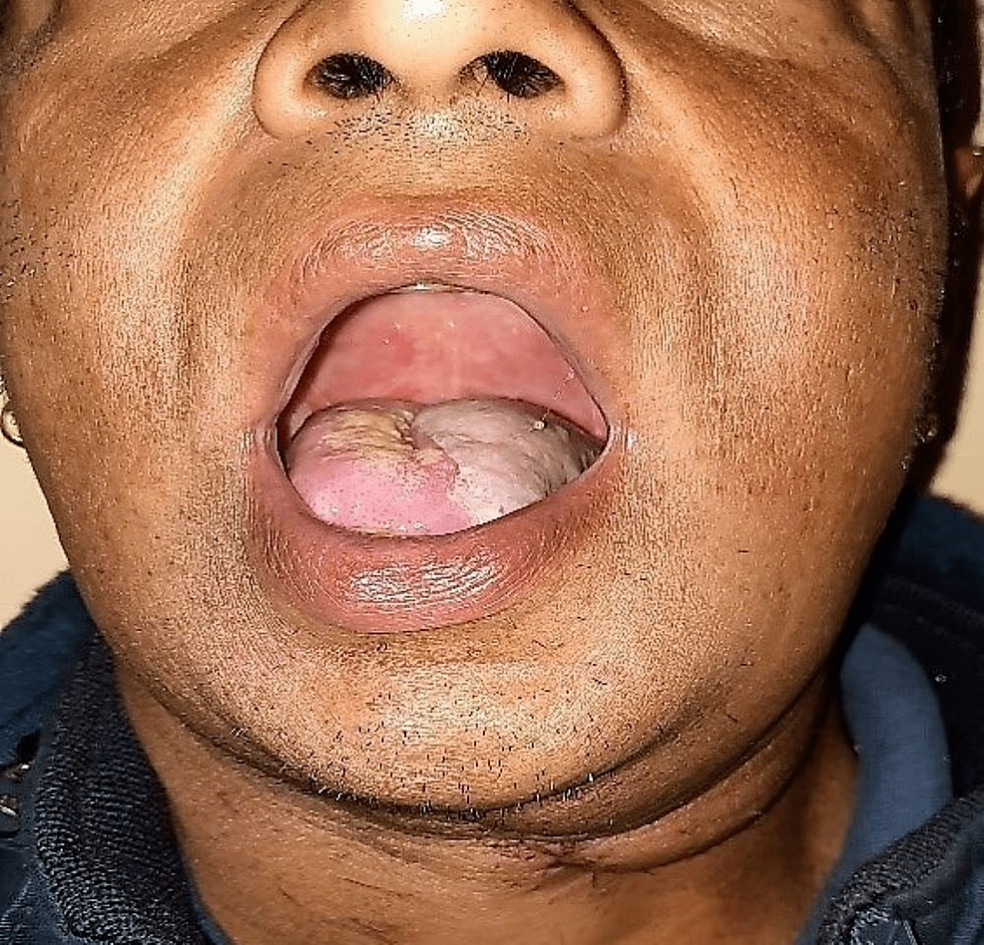
Three-month postoperative photograph.

**Figure 6:**
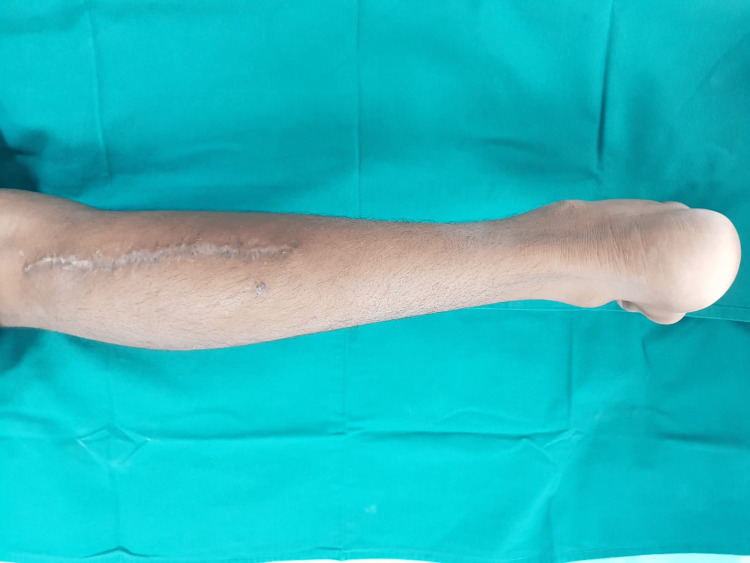
Donor site scar after the three-month follow-up.

The flap was harvested in a semi-froglike supine position, with the knee flexed and the hip abducted. This allows for appropriate exposure to the surgical site, which improves the surgeon’s intraoperative ergonomics. A flap was centered over the marked perforator according to the defect size. However, if a greater pedicle length is raised, the flap can be harvested in an eccentric position, as required. A short 3-4 cm long incision was made parallel to the posterior tibial line and about 2 cm posterior to it. To establish the placement and size of the perforators, the flap’s medial border was lifted. When one or two large perforators were found, the opposing border was incised and the flap was lifted from the medial gastrocnemius muscle with surgical dissection. When elevating the flap to disclose the perforator’s emergence, extreme caution is essential as perforators can be too thin in some situations. The sural nerve and lesser saphenous vein were preserved by elevating the lateral section of the flap. The pedicle was then released by an intramuscular dissection and returned to the popliteal crease until it reached the required length (8-16 cm) or caliber. Bipolar cautery was used to properly perform hemostasis. To prevent vasospasm, papaverine and warm saline were utilized. The length of the vascular pedicle, the number of perforators, and the flap dimensions were all measured. The anastomosis was performed via the facial artery and a tributary of the internal jugular vein (IJV). A Romovac drain was placed for wound drainage. The donor site was closed primarily if the flap was less than 5 cm wide and the tissues were flexible. Otherwise, a skin transplant may be required to avoid compartment syndrome. Split-thickness skin graft (STSG) was treated with a plaster of Paris (POP) splint. Clinical evaluation and handheld Doppler were used to monitor the flap postoperatively. In cases of primary closure donor site cases, patients were mobilized on day one with support, and in STSG cases on day seven (Figures 1-6).

## Results

The average age was 45 years old (range = 32-55). There were 11 men and three women in the study. The average follow-up time was seven months (range = 4-9). Table 1 shows the demographics of the patients. Buccal mucosa (seven cases) and tongue were the most prevalent subsites of the ablative defect (six cases). Twelve of the patients had never received treatment before. Bite marginal composite resection wide local excision of the buccal mucosa with upper alveolectomy and marginal mandibulectomy was the most common procedure performed. Adjuvant radiation was given to 10 patients. Recurrent disease was found in two patients. Adjuvant radiation was given to nine patients.

**Table 1:** Demographic details. FOM: floor of mouth; pT: pathologic tumor stage; M:F: male:female

Age (years)	45.43 (34–55)	
Sex (M:F)	11:3	
Oral cavity subsite: 14	Extent of resection	pT stage (number of cases)
Tongue and FOM: 6	Hemiglossectomy + FOM resection (2)	pT2 (3)
	Anterior two-third glossectomy + FOM resection (4)	pT4 (3)
Buccal mucosa: 7	Bite composite resection (4)	pT3 (3)
	Bite marginal resection (3)	pT4 (4)
Hard palate: 1	Hemipalectomy (1)	pT2 (1)

The average flap measurements were 11.92 (9-15) cm in length, 5.96 (4.5-7) cm in width, and 5.78 (5-7) mm in thickness. Harvesting took an average of 98.57 minutes (75-120 minutes). In our analysis, three large perforators were found in one case, two in four cases, and one in nine cases. The average length of a pedicle was 11.14 cm (8-16 cm). The distance between the major perforator and the popliteal crease on the line connecting the middle of the popliteal crease to the tip of the medial malleolus was 10.025 cm (8-15), and the distance between the major perforator and the posterior midline was 1.475 cm (1-2 cm). In eight cases, the defect was mostly closed, and STSG was harvested in six others.

For anastomosis, the external jugular vein (EJV), IJV tributary, and facial artery were employed. There were two venae comitantes in one case, thus a tributary of the IJV and EJV was chosen for anastomosis. One patient had a neck hematoma, which was removed on postoperative day (POD) two under a local anesthetic. One patient had a flap failure, which was re-examined on POD six. The pedicled pectoralis major myocutaneous flap, on the other hand, was done as a salvageable procedure due to its non-salvageability. In Table 2, the flap parameters are shown. On average, the tracheostomy tube was removed on POD five (4-6), while the Ryles tube was removed on POD eight (6-10). The average length of stay in the hospital was seven (5-15) days.

**Table 2:** Perioperative MSAP flap parameters. *Major perforator distance: point of emergence from the popliteal crease on the line joining from the midpoint of the popliteal crease to the tip of the medial malleolus/posterior midline. **PC→suture dehiscence and infection→STSG. PC: primary closure; STSG: split-thickness skin graft; IJV: internal jugular vein; EJV: external jugular vein; MSAP: medial sural artery perforator

Flap dimensions length (cm) × breadth (cm) × thickness (mm)	Pedicle length (cm)	Major perforator distance from PC/PM (cm)*	Flap harvest time (minutes)	Number of perforators major /minor	Donor-site closure	Anastomosis pedicle artery to	Venae comitantes to	Complications
12 × 6 × 5	12	8.5/1.2	115	1/1	PC	Facial artery	IJV tributary	None
12 × 6.5 × 6	11	8, 12/1, 1.5	120	2/1	PC	Facial artery	IJV tributary	None
13 × 6 × 6	16	8, 12/1, 1.5	90	2/1	STSG	Facial artery	IJV tributary	Neck hematoma
13 × 7 × 6	10	8/2	115	1/1	PC	Facial artery	IJV tributary	None
13 × 6 × 5	8	8/1.4	90	1/1	PC	Facial artery	IJV tributary	None
12 × 6 × 6	10	8/1.1	115	1/1	PC	Facial artery	IJV tributary	None
15 × 7.5 × 5	12	8/1.6	90	1/1	PC→ STSG^**^	Facial artery	Vc1-IJV tributary Vc2-EJV	None
12 × 6 × 5	8	8/2	90	1/1	STSG	Facial artery	IJV tributary	Wound infection
9 × 6 × 7	14	8/1	75	1/1	STSG	Facial artery	IJV tributary	None
11 × 5.5 × 6	10	15/1.4	120	1/1		Facial artery	IJV tributary	Failure
11 × 6 × 6	11	8/1.2	90	1/1	STSG	Facial artery	IJV tributary	None
10 × 5.5 × 5	9	8, 12 /1.6, 1.9	90	2/1	STSG	Facial artery	IJV tributary	None
13 × 5 × 6	10	15, 13, 10 /1.4, 1.9, 2	90	3/0	PC	Facial artery	IJV tributary	None
11 × 4.5 × 7	15	13, 10/1.2, 1.6	90	2/0	PC	Facial artery	IJV tributary	None

The most prevalent donor-site consequence was itching, followed by pigmentation and hypertrophic scarring. In one case, there was suture dehiscence and severe wound infection, as well as transitory functional impairment, which was debrided and controlled with STSG. Speech evaluation was performed on the tongue defects that were rebuilt with MSAP using the speech intelligible rating score. In four cases, grade I (totally normal speech) was achieved, and in two cases, grade II (legible speech to outsiders) was achieved. Table 3 and Table 4 show the functional outcomes and donor-site assessment.

**Table 3:** Donor-site assessment.

Complications	Number of events
Hypertrophic scar	2/14
Itching	5/14
Pigmentation	3/14
Transitory function impairment	1/14
Permanent function impairment	Nil
Donor-site wound dehiscence	1/14

**Table 4:** Functional outcomes. *Follow-up of 12 patients excluding one case of flap failure. **Grade I: completely normal speech; Grade II: legible speech to the outsiders; Grade III: legible to the family only; Grade IV: Illegible speech. POD: postoperative day

Functional outcome variables	Mean - POD	Range - POD
Tracheostomy tube removal*	5	4–6
Ryles tube removal*	8	6–10
Postoperative hospital stay	7	5–15
Follow-up (as on November 1, 2022)	17.093 months**	14.395–19.455 months
Speech assessment in tongue cases (speech intelligible rating score)	Grade I	4
	Grade II	2
	Grade III	Nil

## Discussion

The gold standard for head and neck reconstruction in cancer patients is microsurgical free tissue transfer, which accomplishes both functional and aesthetic restoration. For soft-tissue restoration, the anterolateral thigh flap has become a common option because of its dependability, adaptability, and capacity to harvest a broad skin region with low donor-site morbidity, owing to its long vascular pedicle with an appropriate diameter for anastomosis. Nonetheless, the mass of the flap in medium-sized defects is a substantial disadvantage, particularly in obese individuals, females, or patients with denser subcutaneous fat in the lateral thigh area. In the elevation of the anterolateral thigh perforator flap, Kimura et al. [7] described a debulking approach. However, it can compromise flap circulation or cause direct perforator injury.

Although the radial forearm flap is quite reliable, it has some drawbacks: the sacrifice of a major artery, a cosmetically unpleasant scar at the donor location, and a long healing time in situations of tendon exposure problems. The radial forearm free flap does not give appropriate bulk because it has a relatively thin subcutaneous layer in the forearm area [8,9].

The MSAP flap is a promising choice in this situation, especially in obese individuals and those with small-to-moderate-sized shallow holes. In a single stage, the reconstructive site achieves both cosmetic and functional improvement. The skin of the posterior upper leg is uniformly thin and hairless in appearance. This flap has a muscle-sparing design, resulting in less donor-site morbidity, and its vascular pedicle is lengthy, allowing for several recipient vessel options in the neck. Just like the cephalic vein severing the radial forearm flap, the lesser saphenous vein from the proximal flap boundary can be carefully preserved as another source of venous outflow to the flap. It is possible to work in two teams. When sufficient bulk is required, it can be extracted as a chimeric flap with muscle, medial sural nerve, tendon, or lesser saphenous vein. Because one major branch of the medial sural arteries is usually preserved and multiple secondary vascular pedicles, such as contacts between the lateral and medial heads of the gastrocnemius muscle, are present, the danger of necrosis of the medial gastrocnemius muscle is minimal [10-13].

The donor-site scar and contour distortion remain the biggest drawbacks. Other disadvantages of this flap include the perforator’s unpredictable placement, the search for a reliable perforator, relatively small perforators, and the somewhat time-consuming intramuscular retrograde dissection of the perforator. Furthermore, patients with major lower limb damage, peripheral vascular disease, or diabetes may not be suitable candidates for free MSAP flap transfer. This flap can be used to treat modest-to-medium-sized flaws [13].

The important stage in flap design is finding a competent cutaneous perforator [8]. If the cutaneous perforator is not accurately located, the flap design may be placed outside of the perforator, or the flap size may be changed and increased, obviating the necessity for STSG. Because the perforators may be very small or even non-existent, the MSAP flap is technically demanding. Because the perforators were judged to be too small, Kao et al. [4] had to forgo harvesting three of the 29 flaps in their series. They were not, however, evaluated before surgery, and the authors relied only on anatomical landmarks. In our research, no flaps were abandoned.

Perforator mapping using a handheld Doppler is frequently performed prior to surgery [14]. It is a quick, easy, low-cost, and unreliable method for perforator marking. However, the exact placement of the perforator preoperatively differed from the position of the Doppler perforator in our case series. The Doppler has innovative and necessary clinimetric qualities, according to Stekelenburg et al [14], but it is not suited as a single diagnostic tool for the detection of perforators. Preoperative CT angiography provided accurate and useful information on the recipient vessel's health, perforator location, and caliber.

The flap thickness in our study was between 5 mm and 7 mm, which provided adequate functional and aesthetic adaptation to the oral deformity. As demonstrated in our series, the pedicle length could be as long as 16 cm, which is sufficient to reach the recipient vessels in the neck. With or without a tourniquet, the MSAP flap can be harvested. Without a tourniquet, the operator may better examine the perforator’s pulse, assisting with its assessment. All of our flaps were harvested without the use of a tourniquet in our research. Primary closure is possible without a skin graft in the donor site if the flap width is less than 5 cm.

In our study, the rate of flap failure was 7.14% (1/14), with a late onset on POD seven due to secondary infection. In our research, there was no partial flap collapse. In contrast to our findings, the literature reports a higher rate of flap failure, with the highest rate being 22.2% (2/9) in a study by Toyserkani et al. [15]. In the majority of cases, late-onset flap failures are associated with venous insufficiency, according to the literature. Toyserkani et al. [15] concluded that perforator quality varies, and MSAP with late-onset flap failures is attributable to venous thrombosis. The study found that if only one venous anastomosis is done, the accompanying venae comitantes may be insufficient to drain the flap, and thus provided a viable approach to supercharge the flap to secure venous outflow, which was previously described by Hallock et al. [13]. In all of our patients, a single venous anastomosis was sufficient, and there was no need to supercharge the flap with a saphenous vein in our research. During the harvesting technique, however, extreme caution is used to avoid injuring the perforator and its associated venae comitantes.

The main problems include finding a competent perforator, the time-consuming process of intramuscular retrograde perforator dissection, hair growth of the neo-tongue, and an ugly scar of the skin graft in the donor region [11,12,16,17].

The MSAP flap has a lengthier learning curve than other flaps due to the perforator’s diverse placement and protracted intramuscular dissection, which can be tiresome for the inexperienced.

## Conclusions

The MSAP flap is unusual in that it satisfies both aesthetic and functional requirements in a single procedure while preserving the medial gastrocnemius muscle and major arteries of the leg and reducing donor-site morbidity. It works nicely on the tongue and floor of the mouth, as well as other medium-sized problems. The MSAP flap has a wide range of applications in neck reconstruction, with the benefits of thin and malleable skin, particularly in obese and female patients, a long and dependable vascular pedicle, and minimal donor-site morbidity.
